# Adaptation to reductions in chilling availability using variation in *PLANT HOMOLOGOUS TO PARAFIBROMIN* in *Brassica napus*

**DOI:** 10.3389/fpls.2024.1481282

**Published:** 2024-10-22

**Authors:** Samuel Warner, Carmel M. O’Neill, Rebecca Doherty, Rachel Wells, Steven Penfield

**Affiliations:** Department of Crop Genetics, John Innes Centre, Norwich, United Kingdom

**Keywords:** chilling, vernalization, *Brassica*, temperature, flowering, oilseed rape, climate change

## Abstract

Winter annual crops are sown in late summer or autumn and require chilling to promote flowering the following spring. Floral initiation begins in autumn and winter, and in winter oilseed rape (OSR), continued chilling during flower development is necessary for high yield potential. This can be a problem in areas where chilling is not guaranteed, or as a result of changing climates. Here, we used chilling disruption and low chilling to identify loci with the potential to increase chilling efficiency in winter OSR. We report that time to flowering and yield potential under low chill conditions are affected by variation at the *PLANT HOMOLOGOUS TO PARAFIBROMIN* gene, a component of the plant PAF1c complex. We show that increases in winter chilling given to developing flowers can improve seed yields and that loss of function of *BnaPHP.A05* leads to early flowering in *B. rapa* and *B. napus* and an increase in seed set where chilling is limited. Because *PHP* is known to specifically target the *FLOWERING LOCUS C* (*FLC*) gene in Arabidopsis, we propose that variation at *PHP* is useful for breeding modifications to chilling responses in polyploid crops with multiple copies of the *FLC* gene.

## Introduction

Winter annual crops are widely grown in Europe for their high yields. In the *Brassica* family, winter annual habit is conferred by high expression of orthologs of Arabidopsis *FLOWERING LOCUS C* (*FLC*), which prevent flowering during the longer days of early autumn but are silenced epigenetically in response to prolonged chilling (Tadege et al., 2001; Hou et al., 2012; Yin et al., 2020; Calderwood et al., 2021). *FLC* genes also play a role in the modulation of flowering time in rapid-cycling *Brassica* such as *B. rapa* (Wu et al., 2012). In winter annual *Brassica napus* and in *Arabidopsis thaliana*, vernalization occurs during the lower temperatures of mid-autumn rather than the cold temperatures of winter, followed by floral initiation, which takes place under chilling conditions in the short days of late autumn or early winter (Hepworth et al., 2018; O’Neill et al., 2019; Matar et al., 2021). Silencing of *FLC* genes requires the recruitment of POLYCOMB REPRESSIVE COMPLEX 2 (PRC2) to intron 1 of *FLC* and facultative heterochromatin formation (Questa et al., 2016). On the other hand, active transcription by RNA polymerase II attracts deposition of active epigenetic marks via the activity of the PAF1c complex, which recruits histone methyltransferases to sites of POLII transcription, including *FLC* (Park et al., 2010). For unclear reasons, one subunit of the eukaryotic PAF1c complex known variously as CDC73 or PARAFIBROMIN appears to have a more specialized role in plants and is specifically required for active *FLC* and *FLC*-related gene transcription (Nasim et al., 2022).

After floral initiation, at least two isoforms of *FLC* remain expressed in developing inflorescence meristems in winter oilseed rape (OSR). Further chilling during winter silences these *FLCs*, accelerates flowering, and is also necessary for normal inflorescence development (Lu et al., 2022). In contrast, warming during flower development in winter resulted in a delay to flower bud growth and increased abscisic acid levels indicative of the induction of bud dormancy. Partial fulfillment of the winter chilling requirement is associated with yield declines in a wide range of temperate crops (Atkinson et al., 2013). In the United Kingdom and in China, chilling during early winter is unreliable and reduced chilling correlates with low OSR yields (He et al., 2017; Brown et al., 2019). Climate change is likely to result in a decrease in available winter chill in temperate western Europe and OSR yields globally are vulnerable to changing weather between growing seasons (Rondanini et al., 2012; Campoy et al., 2019), meaning that plant breeding is required to adapt winter OSR to warmer winter environments without compromising yield. Adaptation to lower chill environments requires genotypes that can delay flower initiation until the short days of winter while being reliably chilled at a wider range of chilling temperatures.

Previously, we described a variety trial designed to investigate the genetic control of flowering time in *B. napus* (Lu et al., 2022). In this experiment, a panel of winter, spring, and semi-winter OSR and swedes were grown with sequential sowing such that floral initiation began at the start of winter in Norwich UK in each crop type. The panel was then given control cold winters or a winter warming treatment during flower development, and the effect on flowering time was observed. Our data showed that interrupting flower bud chilling during development initiated a type of winter bud dormancy in lines with high *FLC* expression in reproductive tissues. Here, we use association transcriptomics to further dissect the control of bud dormancy and flowering time in *B. napus* and reveal a role for *PLANT HOMOLOGOUS TO PARAFIBROMIN* (*PHP*) in determining the efficiency of winter chilling in *B. napus*. Because of the known relationship between PHP and *FLC* gene function during chilling in Arabidopsis (Nasim et al., 2022), we further explored its role in bud development in *B. napus* and show that weak *PHP* alleles are associated with increased seed production in laboratory experiments where chilling is limited, suggesting that weak alleles of *PHP* may be useful for crop adaptation to warming winters.

## Results

### Identification of an association between *BnaPHP.A05* genotype and flower bud responses to chilling

**Figure 1:**
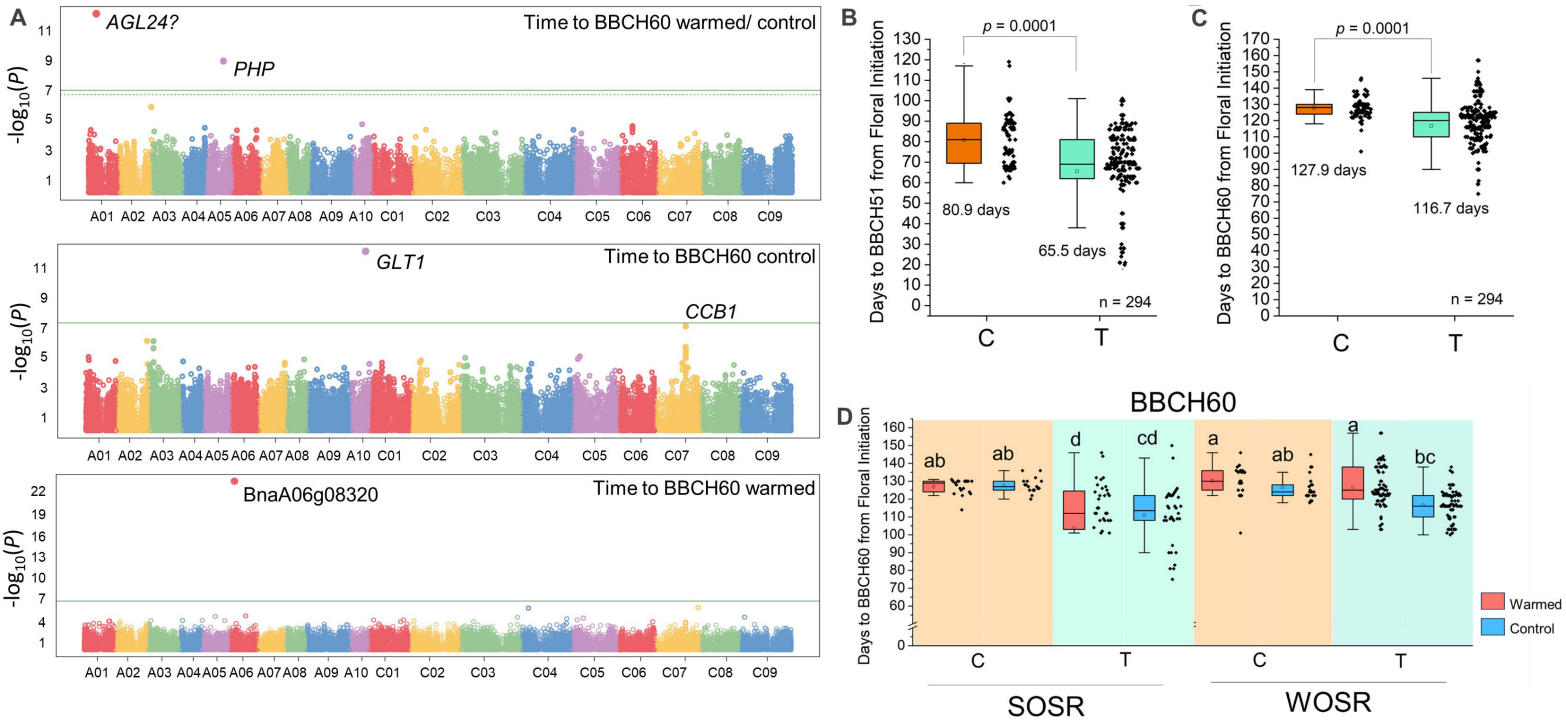
Identification of variation at *BnaPHP.A05* that correlates with the effect of winter temperature variation applied to developing flower buds on flowering time in OSR. **(A)** Manhattan plots reveal loci at which variation correlates with time to BBCH60 (first flower) from floral initiation in either the control winter, warm winter, or the ratio of the two treatments (see Materials and Methods). Significant SNP associations after Bonferroni correction are shown above the horizontal green line. In addition to *BnaPHP.A05*, other loci identified but not analyzed further were orthologs of *A. thaliana AGAMOUS-LIKE 24* (*AGL24*), *GLUCOSE TRANSPORTER 1* (*GLT1*), *COFACTOR OF COMPLEX ASSEMBLY 1* (*CCB1*), and the unknown protein encoded by BnaA06g08320D. **(B, C)** The association between the two identified variants of SNP Cab041204.2:750 (Havlickova et al., 2018) at the *BnaPHP.A05* locus and time from floral initiation to the flowering stages BBCH51 (buds visible; **B**) and BBCH60 (first flowering; **C**) combining flowering data from all the varieties and treatments. Mean times to flowering stages for varieties with each *BnaPHP.A05* SNP call are shown on the charts. **(D)** Time to BBCH60 vs. Cab041204.2:750 SNP call with data from spring and winter OSR in both control and warmed treatments. Significant differences at *p* < 0.05 are indicated using the Tukey *post-hoc* test.

Previous work suggested that effects of winter temperature variation on flower bud development depend on the crop type and are correlated with the *FLC* genotype (Lu et al., 2022). To further understand how temperature variation affects winter OSR flowering behavior, we used Associative Transcriptomics (Havlickova et al., 2018) to uncover SNP variation genetically linked to the effects of flower bud warming on the timing of flowering (**Figure 1A**). We used mean time from floral initiation to BBCH60 in control and warmed treatments plus the ratio between the two measures to uncover loci important on the control of OSR flower bud behavior in response to temperature. From this work, we found several loci with putative associations with flowering time in either the control or warmed treatments, or the effect of temperature on flowering (**Figure 1**). This included one SNP close to *BnaAGL24.A01* on chromosome 1. Exome capture data (Williams et al., 2023) revealed that *BnaAGL24.A01* (BnaA01g13920) has deletions primarily in a small number of Chinese semi-winter lines or unusual spring OSR with fast flowering (**Supplementary Table S1**), but further analysis is complicated by the small number of variant varieties in each crop type in our dataset. A second locus on chromosome A05 had a single SNP associated with the effect of warming on degree days to BBCH60 (**Figure 1A**). Further analysis of the gene implicated on chromosome A05 revealed that this single-nucleotide polymorphism (SNP) is in one of two orthologs of Arabidopsis *PHP*, encoding a component of the Polymerase-Associated Factor 1 complex (PAF1C). PAF1C catalyzes transcription elongation and the interaction of RNA polymerase II with complexes that deposit the active epigenetic marks H3K4 and H3K36 at target gene chromatin, and in so doing promotes active transcription. Loss of PAF1C in Arabidopsis leads to a highly pleiotropic syndrome consistent with widespread disruption in transcriptional control (Nasim et al., 2022). However, PHP is unusual among PAF1C complex proteins in that loss-of-function mutations appear only to reduce *FLC* transcription, causing early flowering in both rapid-cycling and vernalization-requiring Arabidopsis accessions (Yu and Michaels, 2010; Park et al., 2010). *Brassica napus* has two orthologs of the plant *PHP* gene with a second gene found on chromosome C05, but only the A05 gene was implicated in flowering time control. The time from floral initiation to flowering in the control treatment was further associated with two genes with likely roles in plastid function based on homology with Arabidopsis genes, whereas time to flowering in the warmed bud treatment was associated with a gene of unknown function on chromosome A06. These were not followed up in more detail in this study.

Further analysis of *BnaPHP.A05* variation revealed that across the diversity set, lines with the T allele reached BBCH51 a mean of 15.4 ± 2.3 days earlier and first flowering 11.2 ± 2.6 days earlier than lines with the C SNP call (**Figures 1B, C**). The allelic effect showed a significant interaction with crop type, and a much weaker interaction with the effect of warming on different crop types (**Supplementary Table S1**). Because only spring and winter OSR types contained greater than five varieties with both allelic variants (**Supplementary Table S2**), we further analyzed the effect of SNP variation at *BnaPHP.A05* on spring and winter OSR separately. In spring OSR, we found that, on average, varieties with a T SNP flowered substantially earlier than those with a C SNP call, and this was true for both control and warmed plants (**Figure 1D**). Notably, we observed wide variation in flowering time in spring OSR varieties with a T SNP call (**Figure 1D**). In winter OSR, only varieties carrying the T allele were significantly delayed to flowering by the warming treatment applied to developing flower buds, and again, large variation in flowering times was observed in varieties carrying the T allele.

### Haplotype variation at *BnaPHP.A05* and association with time to flowering

**Figure 2:**
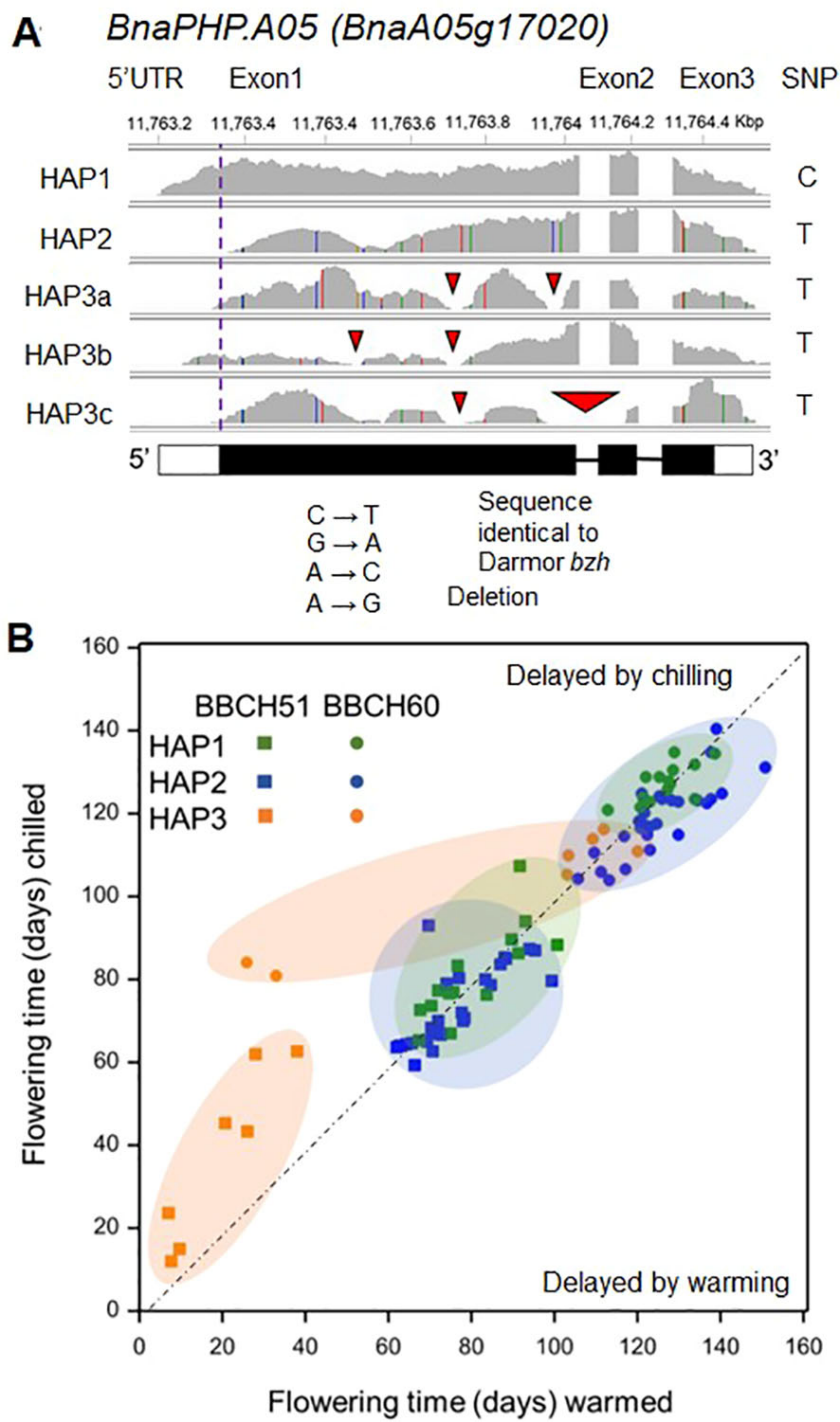
Haplotype variation at *BnaPHP.A05* and its relationship with flowering time in *B*. *napus.* **(A)** Sequence analysis of *BnaPHP.A05* haplotypes aligned to Darmor-*bzh* reference genome revealed that varieties with the Cab041204.2:750:CSNP call resembled Darmor-*bzh*, whereas Cab041204.2:750:T varieties were divided into two categories, HAP2 with a predicted 5′ UTR deletion and HAP3 varieties, which also contained a variety of predicted exon deletions. **(B)** Scatter plot to show the effect of the *BnaPHP.A05* haplotype on mean time to buds visible (BBCH51) or first flowering (BBCH60). Data from Lu et al. (2022). Dashed line divides those varieties accelerated by flower bud warming and those delayed from flower bud warming. An accompanying statistical analysis of the data is shown in **Supplementary Table S2**.

The change from C to T in *BnaPHP.A05* is synonymous and not predicted to affect protein function. To understand how sequence diversity at the *BnaPHP.A05* locus might affect flowering, we analyzed transcriptome sequence data for 48 varieties with either allele including both spring and winter OSR (Havolikova et al., 2019). All varieties with the C *PHP* allele had a *BnaPHP.A05* sequence (HAP1) that matched the Darmor-*bzh* reference sequence (Chalhoub et al., 2014; **Figure 2A**). In contrast, varieties with the T allele showed substantial variation that we broadly categorized into two additional haplotypes. The most frequent, HAP2, contained a large deletion of the putative 5′ untranslated region (UTR): this was up to and included the predicted ATG start codon, suggesting disruption to the PHP protein sequence (we cannot rule out translation of protein variants using alternative start codons). Additional *BnaPHP.A05* variants were found only in spring OSR and combined variable deletions of the 5′ UTR with additional deletions in either the first or the first and second exons (**Figure 2A**). These were grouped into a third heterogeneous haplotype (HAP3) that appears likely to cause loss of function. A small number of SNPs in *BnaPHP.A05* HAP3 genes (**Figure 2A**) mainly led to synonymous amino acid substitutions in the protein sequence; thus, we concluded that deletions were the most likely pertinent variation for *BnaPHP.A05* HAP3. Because the 5′ deletion found in HAP2 and HAP3 could affect promoter activity or transcript stability, we analyzed RNAseq data for *PHP* in the third leaf of the 48 varieties (**Supplementary Figure S1**). HAP2 varieties had a modest 1.5-fold increase in *BnaPHP.A05* expression compared to HAP1 or HAP3 varieties. However, because HAP2 plants share an early flowering phenotype with Arabidopsis *php* mutants, we concluded that changes to the upstream sequences likely do not cause *PHP* gain of function by changing promoter activity or mRNA stability. To understand the contribution of the three haplotypes to flowering time variation, we first compared time from floral initiation to flowering in the control winter and warm winter treatments (**Figure 2**; **Supplementary Table S1**). In winter OSR, HAP1 varieties flowered at a similar time in control and warmed treatments (**Supplementary Table S2**). However, warming caused a significant mean delay of 10 calendar days to flowering in HAP2 varieties. HAP2 varieties were earlier flowering than HAP1 varieties primarily in the control treatment (**Figure 2B**). This suggests that *BnaPHP.A05 HAP2* leads to an enhanced response to chilling in winter OSR. For spring OSR the biggest differences were observed in HAP3 plants containing 5′ UTR and exon deletions. These were earlier flowering than HAP1 and HAP2 regardless of treatment (**Figure 2B**). There was no significant difference in the mean flowering time of HAP1 and HAP2 varieties in spring OSR, unlike in winter OSR. This early flowering phenotype of *BnaPHP.A05* disruption plants resembles that of *php* mutants in rapid-cycling Arabidopsis accessions (Park et al., 2010), raising the prospect that *BnaPHP.A05* loss of function causes early flowering in *B. napus.* Taken together, our analysis shows that HAP2 leads to a greater flowering time sensitivity to chilling than HAP1 in winter OSR.

**Figure 3:**
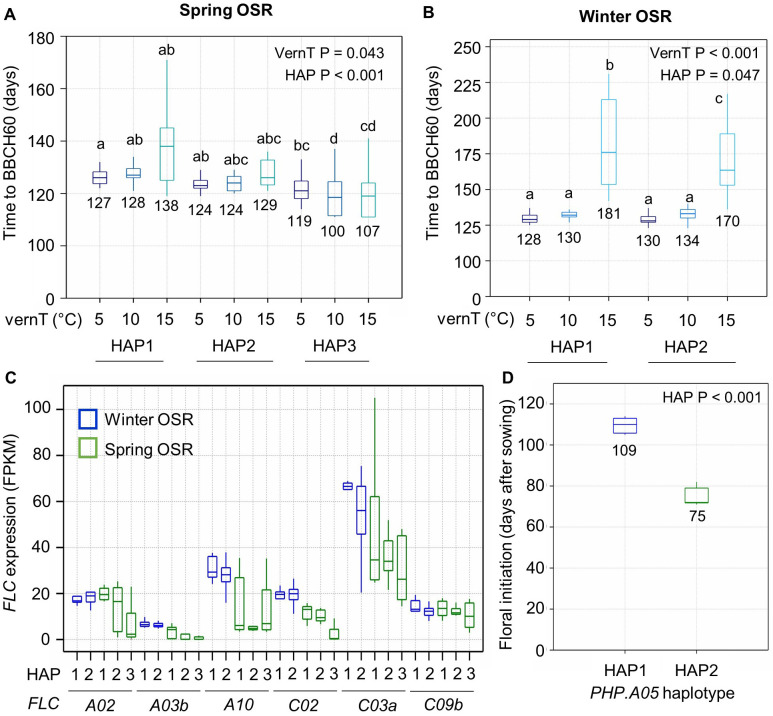
*BnaPHP.A05* variation correlates with vernalization responses and *FLC* expression in *B*. *napus*. **(A)** Boxplot to show the flowering time of spring OSR with three main *BnaPHP.A05* haplotypes after vernalization at three temperatures. *p*-values from two-way ANOVA using vernalization temperature (vernT) and haplotype (HAP) as factors are shown. Significant differences between groups are shown (*p* < 0.05). **(B)** As for A but for winter OSR and the two identified haplotypes of *BnaPHP.A05* in winter oilseed rape varieties. **(C)** Expression of the six highest expressed *B*. *napus FLC* orthologs in the third leaf prior to vernalization. Spring OSR HAP1, *n* = 7; spring OSR HAP2, *n* = 5; and spring OSR HAP3, *n* = 7. Winter OSR HAP1, *n* = 6; winter OSR HAP2, *n* = 21. **(D)** Time for completion of the floral transition from autumn sowing in winter OSR with either *BnaPHP.A05* HAP1 or HAP2. Data indicate the mean and standard error of five plants per genotype. Differences between haplotypes generated by one-way ANOVA.

In a second experiment, we tested whether variation in vernalization temperature between 5°C and 15°C affects time to flowering in a manner that correlates with *BnaPHP.A05* allelic variation (see Materials and Methods). These temperatures are below the upper temperature limit for vernalization but may promote flowering with differing efficiencies (Tommey and Evans, 1991; Wollenberg and Amasino, 2012). In spring OSR, increasing the vernalization temperature from 5°C to 10°C had no significant effect on flowering time, but at 15°C, *BnaPHP.A05* HAP1 and HAP2 plants showed a delay in flowering that varied substantially between varieties (**Figure 3A**). There was no significant difference in flowering time between HAP1- and HAP2-containing varieties, supporting our previous conclusion that HAP2 does not affect flowering time in spring OSR (**Figure 2**). HAP3 varieties flowered earlier than HAP1 and HAP2 varieties in all treatments, and in contrast to HAP1 and HAP2 plants, HAP3 plants flowered earlier at warmer vernalization temperatures. This again suggests that *HAP3* confers earlier flowering.

In winter OSR, increasing the vernalization temperature from 5°C to 10°C again had no significant effect on flowering time and HAP1 and HAP2 varieties showed no significant difference in flowering time. However, 15°C vernalization temperatures conferred a strong delay to flowering that was 11 days longer in HAP1 plants than HAP2 plants, suggesting that in winter OSR, HAP2 confers a more effective chilling response at warmer chilling temperatures (**Figure 3B**). This result supports our previous conclusion that HAP2 is more responsive in flowering time to lower levels of chill.

Vernalization dynamics of OSR depend on the *FLC* genotype, starting *FLC* expression level, and rate of silencing in the falling temperatures of autumn (Schiessl et al., 2019; O’Neill et al., 2019; Calderwood et al., 2021). Because we observed an effect on time to flowering after chilling, we checked for a relationship between *FLC* expression prior to vernalization and *PHP* haplotype, focusing on the most highly expressed *FLC* genes (**Figure 3C**). In spring OSR, while some *FLC* genes were absent or rarely expressed (Schiessl et al., 2019), we found that several orthologs were lower expressed in lines with putative weaker alleles of *PHP*, especially HAP3 plants. This could be because HAP3 causes lower starting FLC levels, but we cannot rule out that the very early flowering of HAP3 lines results in part because of allelic variation at *FLC* in addition to *PHP*. In winter OSR, only *FLC.C03a* had a clear difference between HAP1 and HAP2 plants. To further test whether *BnaPHP.A05*variation affects the speed at which vernalization occurs, we used ecotilling (Woodhouse et al., 2021) to identify varieties of winter OSR with identical *FLC* genotypes but different haplotypes of *BnaPHP.A05* (**Figure 3D**). We found five lines with identical *FLC* genotypes, and a sixth (Castille) with variation in only one *FLC* haplotype at *BnaFLC.C02*: three of these had the putative weak HAP2 and three contained the *A. thaliana*-like HAP1 genotype. Sown in late summer, the HAP2 lines underwent floral initiation a full 34 days prior to the HAP1 lines on average, despite near-identical *FLC* genotypes (**Figure 3D**). While we cannot rule out the presence of other genetic variation that contributes to this affect, this experiment supports the other findings reported here that putative weak alleles of *PHP* have reduced chilling requirements in winter OSR. These observations are consistent with previous observations in *A. thaliana*, which showed that effects of PAF1C disruption on flowering time were stronger in plants subjected to lower temperatures than those maintained at warm temperatures (Yu and Michaels, 2010; Park et al., 2010; Nasim et al., 2022).

### Chilling and weak *BnaPHP.A05* alleles promote gains in seed set

**Figure 4:**
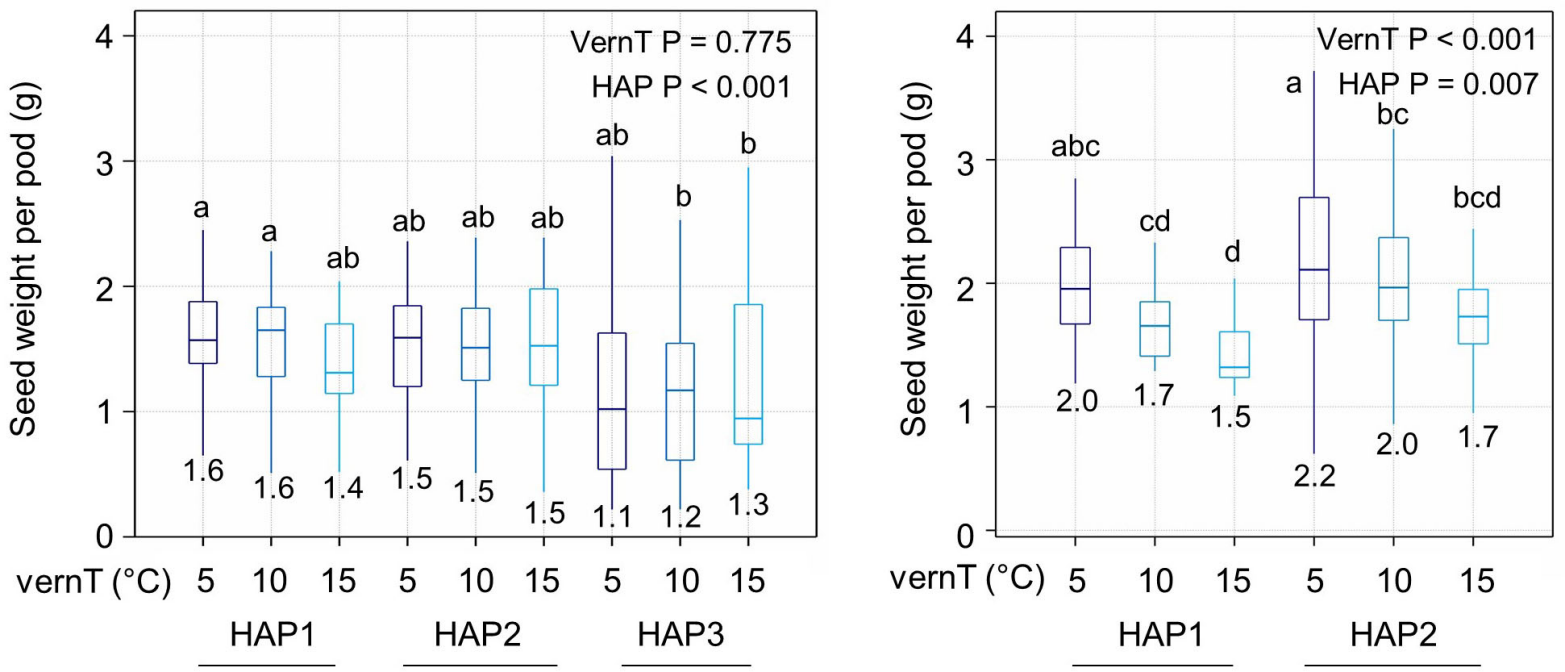
OSR seed yield parameters vary with the *BnaPHP.A05* haplotype. Mean seed weight per pod is shown for lines with each haplotype in each vernalization temperature. *p*-values were calculated by two-way ANOVA with vernalization temperature (vernT) and haplotype (HAP) as factors. Differences between groups are shown at *p* < 0.05. Left plot, spring OSR. Right plot, winter OSR. Mean values for each treatment are indicated on plots. Significant differences are indicated by letters (P < 0.05).

Next, we tested whether changing the vernalization temperature or *BnaPHP.A05* genotype had a relationship with plant yield in winter and spring OSR, measuring seed weight per pod on the primary raceme in plants vernalized at different temperatures (see Materials and Methods). In spring OSR, the vernalization temperature had no discernible effect on seed weight per pod (**Figure 4**). This is not surprising given that spring varieties are known to have a minimal vernalization response. However, HAP3 plants in general had a lower yield than HAP1 and HAP2 plants regardless of the vernalization temperature, perhaps because they are earlier to flower (**Figure 2**). In winter OSR, the picture was strikingly different: firstly, increasing the vernalization temperature resulted in a clear decline in seed weight per pod, regardless of the *BnaPHP.A05* genotype. This shows that in addition to well-known effects of chilling on flowering time and branching, increased chilling also promotes changes to reproductive development that improve seed yield within individual pods. Considering *BnaPHP.A05* variation in winter OSR, HAP2 plants had a 10% higher seed weight per pod (*p* = 0.007) at all tested vernalization intensities, consistent with the enhanced flowering time response to chilling observed in HAP2 varieties. Furthermore, variation at *BnaPHP.A05* can enhance the effect of chilling on seed yield. Thus, we concluded that chilling promotes seed yield in winter OSR and that *BnaPHP.A05* HAP2 increases the yield response to applied chilling within the range tested.

### *PHP.A05* loss of function in *Brassica rapa* causes early flowering

**Figure 5:**
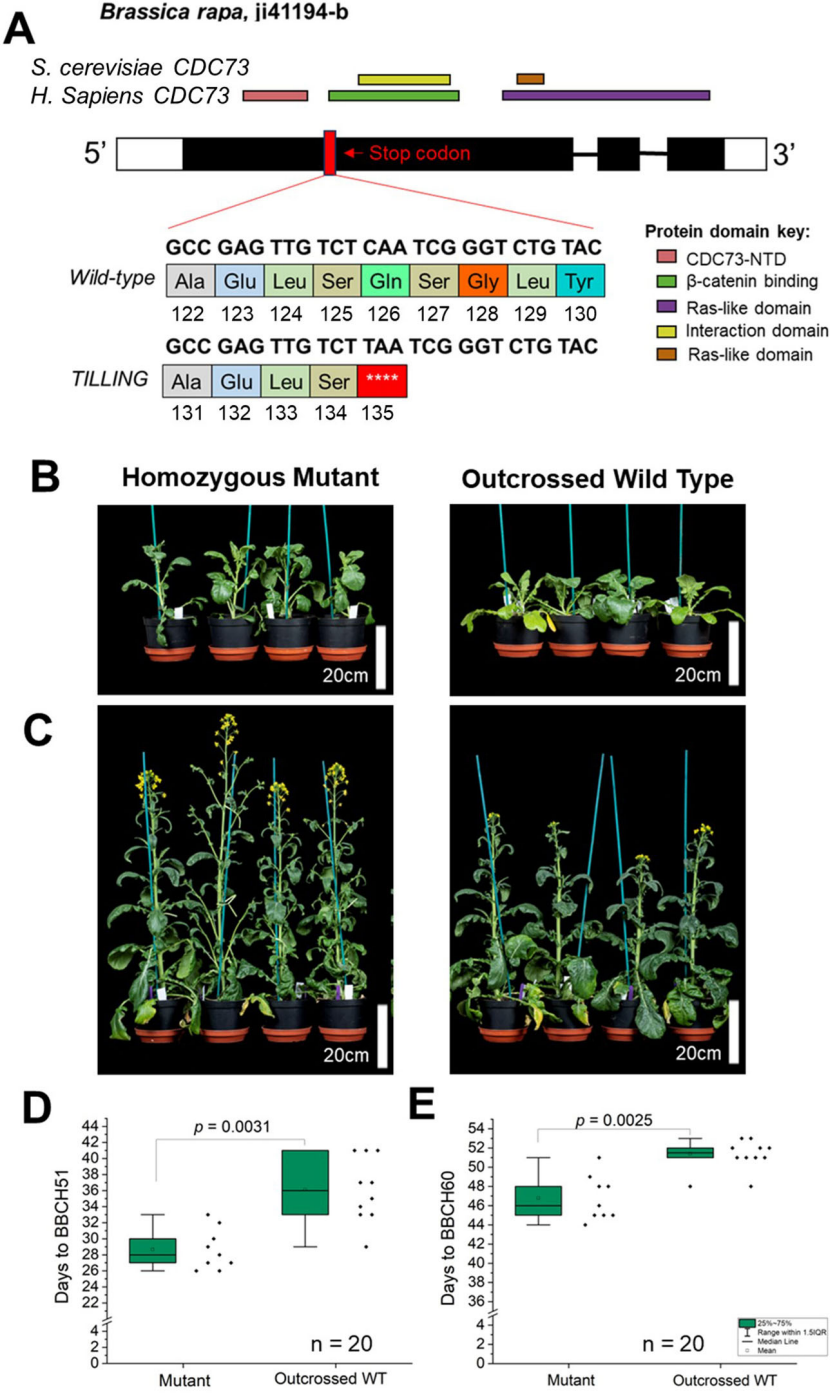
Loss of *BraPHP.A05* function in *Brassica rapa* leads to early flowering. **(A)** Figure to show the structure of the *B*. *rapa PHP.A05* locus and the predicted effect of the tilling mutant ji41194-b on PHP function. **(B, C)** Images to show the flowering time of the *php.a05* mutant and WT segregant. **(D, E)** Plots to show the time to BBCH51 and BBCH60 of WT and *php.a05*, *n* = 10 plants per genotype. Significant differences indicated by one-way ANOVA are shown.

Based on the mutant phenotype reported previously in *A. thaliana*, we assumed that *Brassica* loss-of-function *PHP* alleles would flower early compared to wild type whether in rapid-cycling or vernalization-requiring backgrounds (Yu and Michaels, 2010; Park et al., 2010). To directly test whether variation at *BnaPHP.A05* can cause differences in flowering time, we used a *Brassica rapa* R-o-18 TILLING population (Stephenson et al., 2010) to identify putative loss-of-function alleles of the *BraPHP.A05* gene. *Brassica rapa* shares the *Brassica* A genome with *B. napus*, allowing us to examine the consequences of *PHP.A05* disruption without the complication of tetraploidy. R-o-18 phenology resembles that of rapid-cycling Arabidopsis accessions in that there is no requirement for vernalization for flowering, although there are up to five functional *FLC* orthologs in the species that nevertheless affect time to flowering, as in rapid-cycling Arabidopsis accessions (Wu et al., 2012; Takada et al., 2019; Akter et al., 2023). We found one line, *ji41194-b*, carrying a C-to-T mutation that converted a codon for a glutamine residue at position 186 to a stop codon, a change that should result in premature termination of translation midway through the first exon (**Figure 5A**). From a segregating population, we isolated plants homozygous for the *Braphp.A05* mutant and wild-type alleles and compared their time to flowering. We found that the *Braphp.A05* mutant reached BBCH51 an average of 8 days earlier than wild type and BBCH60 an average of 5 days earlier than wild type in a heated long-day glasshouse (**Figures 5B–E**). Because these differences closely resemble those we found between *B. napus PHP* haplotypes, we concluded that *PHP.A05* loss of function can accelerate flowering across *Brassica* species and crop types.

## Discussion

In Arabidopsis, loss of PHP causes early flowering in vernalization-requiring and vernalization non-requiring backgrounds. In rapid-cycling Arabidopsis, the largest effects of *PHP* on flowering are observed at lower temperatures, approximately 10°C, although PHP affects flowering time at all tested temperatures in Col-0 (Nasim et al., 2022). Our data show that this role appears to be highly conserved in *Brassica* species. We observed that incomplete haplotypes of *BnaPHP.A05* are, in general, associated with either early flowering in spring OSR or an enhanced flowering time response to chilling in winter OSR. We also show that loss of function of the identical gene in *B. rapa* causes a clear early flowering phenotype (**Figure 5**), confirming that in *Brassica* sp., PHP acts to delay flowering as previously observed in Arabidopsis. The observed enhanced response to chilling in weak *php* alleles observed in *B. napus* is also consistent with the known role of PAF1c in delaying flowering via effects on *FLC* chromatin state (Yu and Michaels, 2010).

However, here, we additionally reveal that, in *B. napus*, increases in chilling intensity have additional post-flowering effects on yield components, increasing seed yield within individual pods (**Figure 4**). This yield-promoting effect of chilling is consistent with our earlier observation that interruption to chilling in winter OSR affects crop yields (Brown et al., 2019; Lu et al., 2022). The precise mechanism by which chilling affects yield on a per-pod basis remains to be clarified. Overall, this suggests that in the absence of other considerations, yield gains can be achieved in winter OSR by reducing the vernalization requirement as long as winter annual habit can be maintained. Interestingly, weak *BnaPHP.A05* alleles appear to act additively with chilling, increasing seed yield per pod at all chilling intensities tested (**Figure 4**). PAF1c couples transcription elongation to the deposition of active genetic marks (Krogan et al., 2003; Obermeyer et al., 2022), with this positive feedback important for maintaining the active chromatin state. Our data show that disrupting this feedback can increase the effectiveness of chilling treatments, leading to earlier flowering and higher yields, specifically in winter OSR.

In general, the PAF1c complex appears a poor choice for variation for crop improvement because of its central role in plant chromatin dynamics (Yu and Michaels, 2010). However, PHP appears to be an exception because, in Arabidopsis, its function appears to be limited to the regulation of flowering time via *FLC* expression, with detectable but apparently phenotypically irrelevant roles in the expression efficiency of long transcripts (Park et al., 2010; Nasim et al., 2022; Obermeyer et al., 2022). Because *php* mutants lack the pleiotropic effects of other PAF1c mutants, *PHP* alleles are well suited for adapting OSR crops to lower winter chill or increasing seed yield per pod for which a strong chilling response is advantageous (**Figure 4**). This makes PHP an interesting target for adapting winter OSR to warmer climates, especially maritime Europe where winter chilling is already low in some growing seasons. Fixing weaker *BnaPHP.A05* haplotypes may be useful in winter OSR breeding, especially in adapting the crop to warmer winter environments in maritime Western Europe.

## Materials and methods

### Plant materials

The *B. napus* diversity fixed foundation set (DFFS) has been described previously and was used for the genome-wide association study. This set contains a mixture of winter OSR, spring OSR, Chinese semi-winter OSR, fodder rapes, and swede crop types. *Brassica rapa* R-o-18 and tilling mutant *ji41194-b* were obtained from the Germplasm Resources Unit at the John Innes Centre, Norwich, UK (https://www.seedstor.ac.uk/).

### Genotyping of *B. napus* and *B. rapa* mutants

The presence or absence of the ji41194-b C-to-T mutation in the first exon of *Brassica rapa PHP.A05* that converts glutamine at position 126 to a stop codon was confirmed by PCR from genomic DNA purified using a Macherey-Nagel™ NucleoSpin™ Gel and PCR Clean-up Kit according to the manufacturer’s instructions. *BrPHP.A05* was amplified from *B. rapa* using the forward primer 5′-GTAATCAACGTCCTCTCC-3′ and the reverse primer 5′-GTTGGACCGAATCAGCATAATG-3′, and the presence or absence of the mutation was confirmed by sanger sequencing. The *B. napus* DFFS was genotyped for the SNP at *BnaPHP.A05* with the highest *p*-value (Cab041204.2:750; Havlickova et al., 2018) in *BnaPHP.A05* by PCR amplification from genomic DNA using the forward primer 5′-ATGGATCCGTTATCGGTGCTCAAGG-3′ and the reverse primer 5′-GAACAATTTAACTCACCAGTACTGG-3′ followed by sanger sequencing of the resulting fragment.

### Data

Genotype (SNP) and expression level datasets (Havlickova et al., 2018) were downloaded from York Knowledgebase (http://yorknowledgebase.info) and refined to include only the lines used within this study. Sequence variation in *BnaAGL24.A01* was determined from exome capture data (Williams et al., 2023), NCBI accession number PRJEB57649. *BnaPHP.A05* sequence variation, *BnaPHP.A05*, and *B. napus FLC* expression data were extracted from pre-existing leaf transcriptome data from the Sequence Read Archive accession number PRJNA309367.

### Associative transcriptomics

Associative transcriptomics was performed using the GAGA (GEM and GWAS Automation version 1.0) pipeline (available at https://github.com/bsnichols/GAGA) in R version 4.3.1 for Windows. Association was performed using GAPIT3 and the BLINK multilocus model (Wang and Zhang, 2021; Huang et al., 2019). BLINK runs two fixed-effect models iteratively. The first tests each marker with associated markers fitted as covariates to control for population stratification. The second selects those associated covariate markers and controls for spurious associations that arise in place of kinship (Liu et al., 2016). Loci were selected for further analysis based on significant Bonferroni-corrected *p*-values (Havlickova et al., 2018) and sorting for genes with known roles in flower development. For the further analysis of *BnaPHP.A05*, SNP calls extracted from Havlickova et al. (2018) were tested for a significant correlation with time from floral initiation to flowering using two-way ANOVA. Differences between groups were identified using a Bonferroni *post-hoc* test at *p* < 0.05.

### Vernalization experiments

The *B. napus* DFFS was grown in a heated and lit glasshouse with a daylength of 16 h, a day temperature of 20°C, a night temperature of 15°C for 21 days, and then vernalized for 12 weeks at 5°C, 10°C, or 15°C in controlled environment chambers otherwise matched for light level (80–140 µm m−2 sec−1) in 8-hour days. After vernalization, plants were potted into 1-L pots and maintained in the long-day glasshouse with a temperature of 18°C for flowering and seed set. Flowering time was scored using the BBCH scale (Weber and Bleiholder, 1990). Yield per plant was estimated by analyzing seed number, size, and weight from 20 filled pods from the primary raceme, as described previously (Miller et al., 2019). Mean flowering time for each variety was used for statistical association with the *BnaPHPA05* haplotype using two-way factorial ANOVA. Differences between groups were identified using a Bonferroni *post-hoc* test at *p* < 0.05.

In a second experiment to monitor the timing of floral initiation after vernalization, six varieties of winter OSR were grown, Rafal, Dippes, and Eurol with *PHP.A05* HAP1, and Castille, Temple, and Rameses with *PHP.A05* HAP2, in an unheated unlit glasshouse with seeds sown at the beginning of autumn (September 2022). These lines had identical *FLC* haplotypes, except for Castille, which has a homoeologous exchange of *FLC.C02* for an extra copy of *FLC.A02*. *FLC* haplotype data have been described previously (Lu et al., 2022). Plants were monitored for the completion of vernalization by the timing of floral initiation using manual dissection under a Leica SP6 dissecting microscope.

### Winter warming experiment

The *B. napus* DFFS was sown in an unheated unlit polytunnel, with timing of sowing staggered such that all varieties initiated flowers in November in Norwich, UK. Plants were maintained in the polytunnel over winter or moved 1 week after flowering to an unlit glasshouse at 20°C for 4 weeks. Precise details of temperature treatments were monitored using Gemini Tinytag Plus 2 TGP-44017 temperature and humidity loggers and are shown in **Supplementary Figure S2**. More details have been given previously (Lu et al., 2022). These conditions approximately match known field conditions in the UK in high- and low-yielding growing seasons (**Supplementary Figure S3**). After 4 weeks, all plants were returned to the unheated polytunnel, randomized, and maintained until flowering. Flowering time was scored on the BBCH scale.

## Data Availability

Genotype (SNP) and expression level datasets (Havlickova et al., 2018) were downloaded from York Knowledgebase (http://yorknowledgebase.info) and refined to include only the lines used within this study. Sequence variation in *BnaAGL24.A01* was determined from exome capture data (Williams et al., 2023), NCBI accession number PRJEB57649. *BnaPHP.A05* sequence variation, *BnaPHP.A05* and *B. napus FLC* expression data were extracted from pre-existing leaf transcriptome data from the Sequence Read Archive accession number PRJNA309367.
