# Supplementary material for: Adaptation to reductions in chilling availability using variation in PLANT HOMOLOGOUS TO PARAFIBROMIN in Brassica napus
Source: Front Plant Sci. 2024 Oct 22;15:1481282. doi: 10.3389/fpls.2024.1481282 (PMC11534679; doi:10.3389/fpls.2024.1481282)
Supplement: Supplementary file 1 [file DataSheet1.docx]

Supplementary Material

# Supplementary Tables

**Table S1. P values from two-way factorial ANOVA comparing the effect of *PHP.A05* haplotype on the response to chilling interruption during flower bud development.** See Figure 2 for more details. Spring and winter OSR were analysed separately because HAP3 is unique to spring varieties, and because lines are bred for different chilling responses. Asterisks indicate significant effects at the P < 0.01 (*) and 0.001 (**).

| **BBCH51 WOSR** | **Degrees of freedom** | **Sum of squares** | **Mean square** | **Variance ratio** | **P value** |
| --- | --- | --- | --- | --- | --- |
| haplotype | 1 | 861.3 | 861.3 | 7.72 | 0.007* |
| treatment | 1 | 147 | 147 | 1.32 | 0.256 |
| haplotype. treatment | 1 | 82.6 | 82.6 | 0.74 | 0.393 |
| **BBCH60 WOSR** |  |  |  |  |  |
| haplotype | 1 | 425.13 | 425.13 | 5.26 | 0.026* |
| treatment | 1 | 1046.21 | 1046.21 | 12.95 | < 0.001** |
| haplotype. treatment | 1 | 103.32 | 103.32 | 1.28 | 0.263 |
| **BBCH51 SOSR** |  |  |  |  |  |
| haplotype | 2 | 18563.8 | 9281.9 | 61.05 | < 0.001** |
| treatment | 1 | 535.6 | 535.6 | 3.52 | 0.07 |
| haplotype. treatment | 2 | 646.7 | 323.3 | 2.13 | 0.136 |
| **BBCH60 SOSR** |  |  |  |  |  |
| haplotype | 2 | 8100.6 | 4050.3 | 11.03 | < 0.001** |
| treatment | 1 | 230.9 | 230.9 | 0.63 | 0.434 |
| haplotype. treatment | 2 | 570.9 | 285.5 | 0.78 | 0.468 |

**Table S2. Haplotype scores of *Bna.AGL24-A01* by crop type in *B. napus***. Haplotypes were scored using exome capture sequence data for flowering time-associated genes (Woodhouse et al., 2021). Haplotype 1 is identical to the darmor BZH reference sequence, HAP2 contains miscellaneous SNPs whereas haplotype 3 contains a clear deletion, based on no recovery of reads.

| **Haplotype** | **crop type** | **number of varieties** |
| --- | --- | --- |
| 1 (Darmor) | spring OSR | 18 |
| 2 | spring OSR | 2 |
| 3 (deletion) | spring OSR | 1 |
| 1 (Darmor) | winter OSR | 37 |
| 2 | winter OSR | 1 |
| 3 (deletion) | winter OSR | 0 |
| 1 (Darmor) | semi-winter OSR | 5 |
| 2 | semi-winter OSR | 0 |
| 3 (deletion) | semi-winter OSR | 3 |
| 1 (Darmor) | swede | 7 |
| 2 | swede | 2 |
| 3 (deletion) | swede | 0 |
| 1 (Darmor) | Exotic/fodder | 14 |
| 2 | Exotic/fodder | 1 |
| 3 (deletion) | Exotic/fodder | 1 |
| 1 (Darmor) | All | 81 |
| 2 | All | 6 |
| 3 (deletion) | All | 5 |


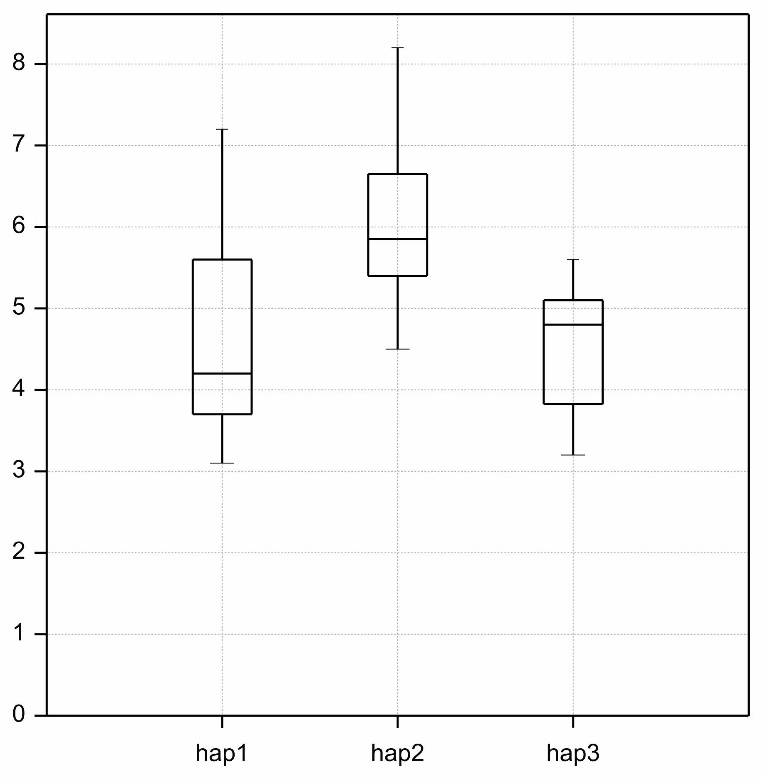


HAP1

HAP2

HAP3

0

1

2

3

4

5

6

7

8

a

a

b

*BnPHP-A05* expression *(*tpm*)*

*BnaPHP.A05* genotype

P < 0.001

n = 14

n = 7

n = 27

**Figure S1. Expression of *BnaPHP.A05* in the transcriptome of the third leaf in 48 varieties of winter and spring *B. napus* for which the *BnaPHP.A05* haplotype was determined.** Significant differences were revealed by one-way ANOVA. Number of varieties with each haplotype in the dataset is shown. Data extracted from Havlickova et al., (2018).


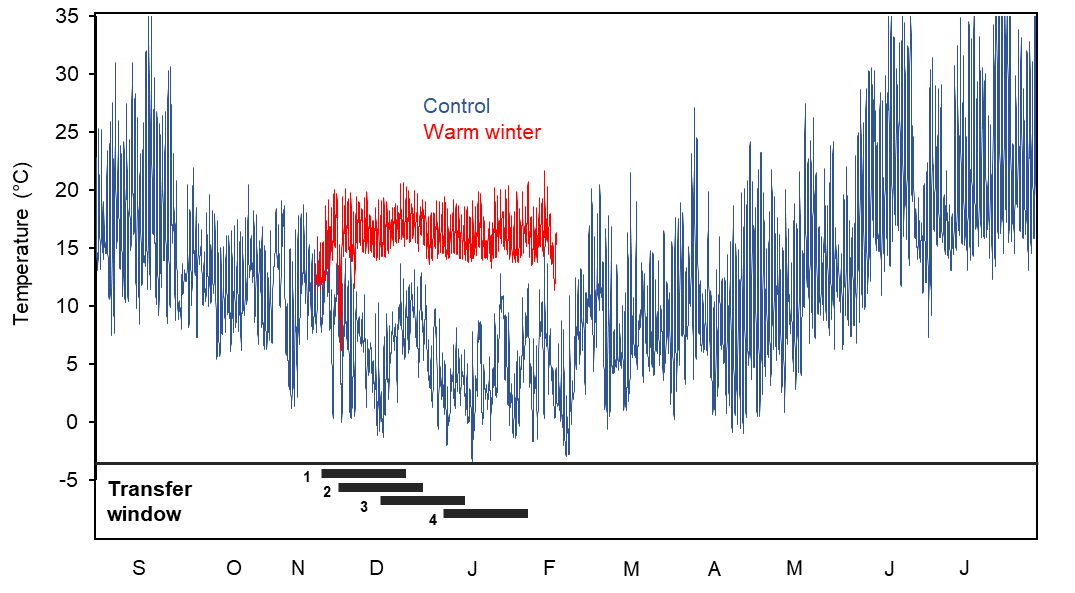


**Figure S2. Temperature treatments given to the *Brassica napus* diversity set**. Plants were grown in an un-heated unlit polytunnel (blue) until floral initiation. After floral initiation plants were either maintained in the polytunnel (control treatment) or transferred to a warmed but unlit glasshouse (red) for a period of four weeks. Variation in time to floral initiation among varieties resulted in transfers being undertaken in four cohorts. At the end of the four week treatments all plants were maintained in the polytunnel, randomized and allowed to flower.

**Figure S3. Example climate histories of two UK winter annual growing seasons with contrasting winter OSR yields.** 2010/11 was a high yielding year with a cold early winter (blue temperature series) and 2015/16 a low yielding year with a warm early winter. Data taken from Brown et al. (2019) using daily temperature data from the Hadley Centre Central England Temperature (HADCETII) series.
